# Analysis of Differentiated Chemical Components between Zijuan Purple Tea and Yunkang Green Tea by UHPLC-Orbitrap-MS/MS Combined with Chemometrics

**DOI:** 10.3390/foods10051070

**Published:** 2021-05-12

**Authors:** Mengwan Li, Ying Shen, Tiejun Ling, Chi-Tang Ho, Daxiang Li, Huimin Guo, Zhongwen Xie

**Affiliations:** 1 State Key Laboratory of Tea Plant Biology and Utilization, School of Tea and Food Sciences and Technology, Anhui Agricultural University, Hefei 230036, China; mengwanli@ahau.edu.cn (M.L.); Yshen74@its.jnj.com (Y.S.); lingtj@ahau.edu.cn (T.L.); dxli@ahau.edu.cn (D.L.); 2 International Joint Laboratory on Tea Chemistry and Health Effects of Ministry of Education, School of Tea and Food Sciences and Technology, Anhui Agricultural University, Hefei 230036, China; ctho@sebs.rutgers.edu; 3 Department of Food Science, Rutgers University, 65 Dudley Road, New Brunswick, NJ 08901, USA; 4 Center for Biotechnology, Anhui Agricultural University, Hefei 230036, China

**Keywords:** Zijuan purple tea, Yunkang green tea, metabolite profiling, UHPLC–Orbitrap–MS/MS, characteristic chemical compounds, anthocyanins, proanthocyanins

## Abstract

Zijuan tea (*Camellia sinensis var. assamica* cv. Zijuan) is a unique purple tea. Recently, purple tea has drawn much attention for its special flavor and health benefits. However, the characteristic compounds of purple tea compared with green tea have not been reported yet. The present study employed a non-targeted metabolomics approach based on ultra-high performance liquid chromatography (UHPLC)-Orbitrap-tandem mass spectrometry (MS/MS) for comprehensive analysis of characteristic metabolites between Zijuan purple tea (ZJT) and Yunkang green tea (YKT). Partial least squares-discriminant analysis (PLS-DA) indicated that there are significant differences in chemical profiles between ZJT and YKT. A total of 66 major differential metabolites included catechins, proanthocyanins, flavonol and flavone glycosides, phenolic acids, amino acids and alkaloids were identified in ZJT. Among them, anthocyanins are the most characteristic metabolites. Nine glycosides of anthocyanins and six glycosides of proanthocyanins were found to be significantly higher in ZJT than that in YKT. Subsequently, pathway analysis revealed that ZJT might generate anthocyanins and proanthocyanins through the flavonol and flavone glycosides. Furthermore, quantitative analysis showed absolutely higher concentrations of total anthocyanins in ZJT, which correlated with the metabolomics results. This study presented the comprehensive chemical profiling and the characterized metabolites of ZJT. These results also provided chemical evidence for potential health functions of ZJT.

## 1. Introduction

Tea plants (*Camellia sinensis* (L.) O. Kuntze) are widely distributed in southwestern China and have been used to manufacture various tea types [1]. Because of beneficial effects for human health, tea has become a worldwide consumed beverage [2,3]. During long-term natural hybridization and under human selection pressure, the leaf color of tea plants has greatly diversified. The selection of specific colors of tea leaves has been applied in recent breeding programs. Zijuan (*C. sinensis var. assamica* cv. Zijuan) is the first registered purple tea cultivar with outstanding purple buds, stems and leaves. During recent years, the leaves of Zijuan cultivar have been used to produce purple tea, which are very popular for purple colors and unique flavors.

It is worth noting that Zijuan and Yunkang tea cultivar (*C. sinensis var. assamica* cv. Yunkang) are both classified as the same taxonomic variant of *C. sinensis var. assamica*, and both originated from the Yunnan province of China. Following a standard procedure of fixation, rolling and drying, the leaves and buds of Zijun and Yunkan are processed into green teas. Green tea is an unoxidated tea type conserved highest amount of original chemical compounds in the leaves and buds of tea plants. Accumulating evidence showed that green tea extract (GTE) has many health benefits, such as antibacterial, anti-obesity, anti-hyperglycemic, antioxidant and anticancer activity. Zijun purple tea (ZJT) presents a special aroma and purple color compared with Yunkang green tea (YKT). Some research reported that ZJT contains abundant anthocyanins, which are naturally occurring pigments accounting for the purple, red and blue colors of many plant organs. Jiang et al. successfully identified four unique anthocyanins from ZJT. Among them, delphinidin-3-*O*-beta-D-galactoside and cyanidin-3-*O*-beta-D-galactoside were confirmed by liquid chromatography−electrospray ionization mass spectrometry (LC-ESI-MS) [4]. Wang et al. also reported some new compounds from ZJT, which are delphinidin-3-*O*-beta-D-(6-(E)-*p*-coumaroyl) galactopyranoside and cyanidin-3-*O*-beta-D-(6-(E)-*p*-coumaroyl) galactopyranoside identified by the high-resolution time-of-flight-mass spectrometry (TOF-MS) and nuclear magnetic resonance (NMR) techniques [5,6]. Published papers reported that ZJT has pharmacological activities [7,8], and the large molecular polymeric pigments isolated from ZJT significantly reduce blood lipids in high-fat diet feeding rats. Additionally, ZJT executes antihypertensive effect for its rich in flavonoids, zinc and anthocyanins [6,9]. However, the metabolite profiling and characteristic chemical composition compared with green tea has not been reported yet.

Non-targeted metabolomics, aiming at comprehensive chemical profiling, has been playing a pivotal role in quality control, species differentiation and processing mechanistic investigation [10,11,12,13]. It has been widely used in tea classification [14], tea processing [15,16], tea origins [17], and picking time [2,18,19]. Comparing with various profiling techniques in plant metabolomics, the ultra-high performance liquid chromatography–tandem mass spectrometry (UHPLC-MS/MS) technique offers remarkable advantages in resolution, sensitivity, and analysis speed. Orbitrap is one of widely used mass spectrometry technique in metabolomics research given its capacity for a non-targeted search and fragmentation [20]. In this study, a non-targeted metabolomics approach utilizing UHPLC-Orbitrap-MS/MS was established for comparatively analyzing ZJT and YKT, with metabolite profiling and chemometrics. Our results will provide the first set of data for better understand chemical profiles and characteristic compounds between ZJT and YKT, and also provide chemical information for potential health benefits of ZJT.

## 2. Materials and Methods

### 2.1. Chemicals and Tea Samples 

LC-MS-grade water, acetonitrile and methanol were bought from CNW Technologies GmbH (Düsseldorf, Germany). Formic acid of LC-MS grade was provided by General Electric Company ((Fairfield, CT, USA). Ammonium hydrogen carbonate was obtained from Thermo Fisher Scientific (Sunnyvale, CA, USA). Catechin (C, purity ≥ 98%), epicatechin (EC, purity ≥ 98%), gallocatechin (GC, purity ≥ 98%), epigallocatechin (EGC, purity ≥ 98%), epicatechin gallate (ECG, purity ≥ 98%), catechin gallate (CG, purity ≥ 98%), epigallocatechin gallate (EGCG, purity ≥ 99%) and caffeine (purity ≥ 98%) were obtained from Chengdu MUST Bio-technology Co., Ltd. (MUST, Chengdu, China). The steamed tea Zijuan purple tea (ZJT) and Yunkang green tea (YKT) were produced in 2017 by Yunnan Tea Science Research Institute. The fresh leaves of Zijuan cultivar (*C. sinensis var. assamica* cv. Zijuan) and Yunkang cultivar (*C. sinensis var. assamica* cv. Yunkang) were picked at the same time from the tea garden of the Institute of Tea Science, Yunnan Province Academy of Agricultural Sciences (Menghai, China), respectively. They were processed into green tea using typical manufacturing approached. Briefly, the leaves were fist steamed at 220 °C, then rolled for 30 min, and finally dried by electrical heaters into green tea. The vouchers of the Zijuan and Yunkang plants were preserved in the Institute of Tea Science, Yunnan Province Academy of Agricultural Sciences.

### 2.2. Sample Preparation

Each powdered sample (80 mg) was extracted with 1.0 mL of methanol/water (70:30, *v*/*v*) for 10 min at 4 °C under 100 Hz ultrasonic extraction. After centrifuging at 14,000 rpm for 10 min, the supernatants were diluted 40-fold with methanol/water (70:30, *v*/*v*) and then filtered through 0.22 μm nylon filter for MS analysis. A 5 μL aliquot of the extract was injected into UHPLC-Orbitrap-MS/MS. Quality control samples were obtained by mixing equal volumes of each sample analyzed in the present investigation to monitor system stability in non-targeted metabolomics.

### 2.3. Ultra-High Performance Liquid Chromatography Combined with Orbitrap Mass Spectrometry (UHPLC-Orbitrap-MS/MS) Analysis

Chromatographic analysis was carried out on an Ultimate HPLC system (Thermo Fisher Scientific, Waltham, MA, USA). In a positive ion mode, a small volume of 5 μL sample was separated on an ACQUITY BEH C18 column (100 mm × 2.1 mm, 1.7 μm; Waters, Milford, MA, USA), which temperature was held at 35 °C. A (0.1% formic acid in water) and B (0.1% formic acid in acetonitrile) were used as mobile phase. And the flow rate was set at 0.35 mL/min under gradient elution conditions: 0–1 min, 95% A; 1–24 min, 95% A; 24–28 min, 0% A. Then the column re-equilibration step was performed at initial composition in 1 min and held for another 3 min. In a negative ion mode, samples were separated with an ACQUITY HSS T3 column (100 mm × 2.1 mm, 1.8 μm; Waters, Milford, MA, USA). The elution system comprised A (6.5 mM ammonium acetate in water) and B (6.5 mM ammonium acetate in 95% acetonitrile), and the solvent gradient was set as follows: 0–1 min, 95% A; 1–18 min, 95–0% A; 18–22 min, 0% A; 22.1–25 min, 0–95% A; 25–28 min, 95% A.

MS detection was executed on a Q Exactive Focus Oribitrap MS system (Thermo Fisher Scientific, USA) supplied with a heated electrosprayer for ionization (HESI). Mass spectra were acquired separately with positive and negative ionization mode in a full mass operation with a mass range of 100–1000 m/z by the spray voltage of 3.5 and 3 kV respectively. The temperature of capillary and source were maintained at 350 and 360 °C. The pressure of 50 psi and 10 psi was set for sheath gas (N_2_) and auxiliary gas (N_2_), respectively. Sodium trifluoroacetate was used for accurate mass calibration. Mass spectra and chromatograms were acquired and processed with Xcalibur (Thermo Fisher Scientific, Waltham, MA, USA). Chemical formula for precursor and product ions was determined by software of Compound Discoverer (version 2.0).

### 2.4. Catechins and Caffeine Analysis by HPLC

The concentration of catechins and caffeine was analyzed by HPLC (Waters, Milford, MA, USA). The protocol for chromatographic performance were slightly modified from a previous publication [21]. Briefly, chromatographic separation was executed on a Phenomenex Gemini C18 column (100 mm × 4.6 mm, 5 μm; Torrance, CA, USA). A small volume of 5 µL sample was loaded on the column, which maintained at 25 °C. Then, the sample was eluted at the rate of 1 mL/min, and was detected at the wavelength of 278 nm, respectively. The mobile phase A (deionized water with 0.17% acetic acid) and mobile phase B (100% acetonitrile) were used for the linear gradient, which was set as follows: mobile phase B from 8–28.4% (*v*/*v*) in 30 min was initiated, from 28.4–100% (*v*/*v*) for 8 min, and from 100–8% (*v*/*v*) for another 10 min at a flow rate of 1.0 mL/min.

### 2.5. Total Content of Anthocyanins by Acid-Alcohol Method

Total content of anthocyanins was determined following the method of Jiang et al. [22] with slight modification. Briefly, two gram of tea powdered sample was extracted with 40 mL boiling water for 30 min. The obtained extracts were centrifuged at 3500 rpm for 10 min, and were further diluted with 1% acidic ethanol to a final volume of 50 mL. The absorbance value of the extracts was measured at 535 nm.

### 2.6. Data Analysis for Untargeted Metabolomics

The raw UHPLC-Orbitrap-MS/MS data were progressed by XCMS Online (http://xcmsonline.scripps.edu/, accessed on 16 December 2020) [23] for peak deconvolution and alignment. The primary parameters were set as follows: 100,000 for ion intensity threshold, 20 ppm for ion m/z tolerance, 0–30 min for retention time range, and 0.5 min for ion retention time tolerance. Other parameters were set as default. Area method was used to normalize the data. Finally, with the established methods [24,25], a data matrix was obtained and exported to an Excel table, including retention time (RT), m/z value and normalized ion intensity (variables).

The preprocessed data sets from positive and negative models were imported into SIMCA-P software (version 13.0) (Umetrics, Umeå, Sweden) to perform unsupervised principal component analysis (PCA) and supervised partial least squares discriminant analysis (PLS-DA). The variable importance in the projection (VIP) value was calculated in the PLS-DA model, *p*-value was determined in non-parametric tests (Mann–Whitney test), and fold change (FC, ZJT/YKT) was compared between ZJT and YKT. The variables with VIP > 1.0, *p*-value < 0.05 and |log_2_−FC| > log_2_ 1.5 were chosen as potential differential metabolites [26].

The structure annotation of the potential differential metabolites was determined by comparing RT, m/z with that from published literature [14,17,27,28,29], such as OSI/SMMS (jointly developed by Dalian Institute of Chemistry and Physics and Dalian Dashuo Information Technology Co., Ltd., Dalian, China), HMDB (http://www.hmdb.ca/, accessed on 12 February 2021) [30], PubChem (http://www.ncbi.nlm.nih.gov/pccompound/, accessed on 20 January 2021) [31], METLIN (http://metlin.scripps.edu/, accessed on 20 January 2021) [32], and Tea Metabolism Database (TMDB, http://pcsb.ahau.edu.cn:8080/TMDB/teaAction_search.action, accessed on 20 January 2021) [33]. The KEGG Pathway Database (http://www.genome.jp/kegg/pathway.html, accessed on 20 January 2021) [34] was employed for pathway analysis.

## 3. Results

### 3.1. Significant Differentiation of Zijuan Purple Tea (ZJT) and Yunkang Green Tea (YKT) Metabolite Profiling by UHPLC-Orbitrap-MS/MS

**Figure 1:**
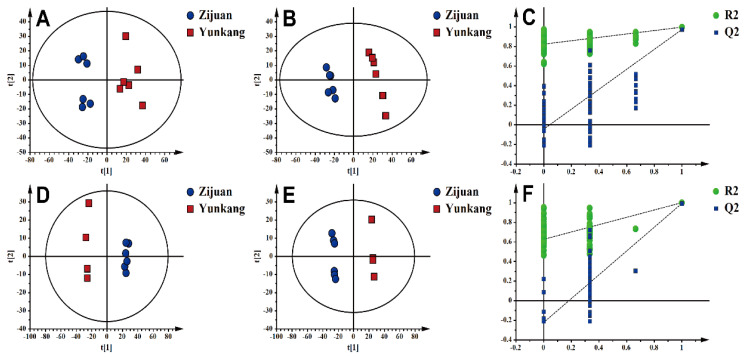
Multivariate statistical analysis of the differences in metabolites between Zijuan purple tea (ZJT) and Yunkang green tea (YKT). (**A**) Principal component analysis (PCA)-X score in positive ion mode (R^2^X = 0.73; Q^2^ = 0.47); (**B**) partial least squares-discriminant analysis (PLS-DA) score in positive ion mode (R^2^X = 0.519; R^2^Y = 0.996; Q^2^ = 0.972); (**C**) PLS-DA model validation in positive ion mode (R^2^ = 0.818; Q^2^ = −0.0476); (**D**) PCA-X score in negative ion mode (R^2^X = 0.638; Q^2^ = 0.506); (**E**) PLS-DA score in negative ion mode (R^2^X = 0.656; R^2^Y = 0.999; Q^2^ = 0.991); (**F**) PLS-DA model validation in negative ion mode (R^2^ = 0.626; Q^2^ = −0.226).

The metabolites in the ZJT and control green tea (YKT) were analyzed with the UHPLC-Orbitrap-MS/MS system. The typical total ion current chromatograms of tea samples were analyzed in positive and negative ion modes (Figure S1), which yielded 1635 and 1587 metabolite ion features, respectively, for statistical analyses. At first, to identify intra- and inter-class differences between ZJT and YKT, we conducted an unsupervised PCA analysis. Our results showed that the samples were closely clustered as ZJT and YKT groups, and the quality control (QC) samples gathered together, reflecting the stability and reliability of the metabolomics analysis. Furthermore, the ZJT and YKT samples were clearly separated, indicating a significant difference in chemical profiles between ZJT and YKT in positive and negative ion mode (Figure 1A,D).

The supervised PLS-DA model, which produced analogous results, was applied to examine the metabolites with the greatest differences. The ZJT samples were clustered within II and III quadrants, and the YKT samples were separated with ZJT samples in I and IV quadrants. These results further confirmed that ZJT and YKT have distinct chemical profiles (Figure 1B,E). Furthermore, R^2^X, R^2^Y and Q^2^ were calculated as 0.519, 0.996 and 0.972 in positive and 0.519, 0.996, 0.991 in negative, respectively, which indicated that the models were established successfully without over-fitting [35]. Permutation had the same results (Figure 1C,F).

### 3.2. Characteristic Metabolites of ZJT

**Figure 2:**
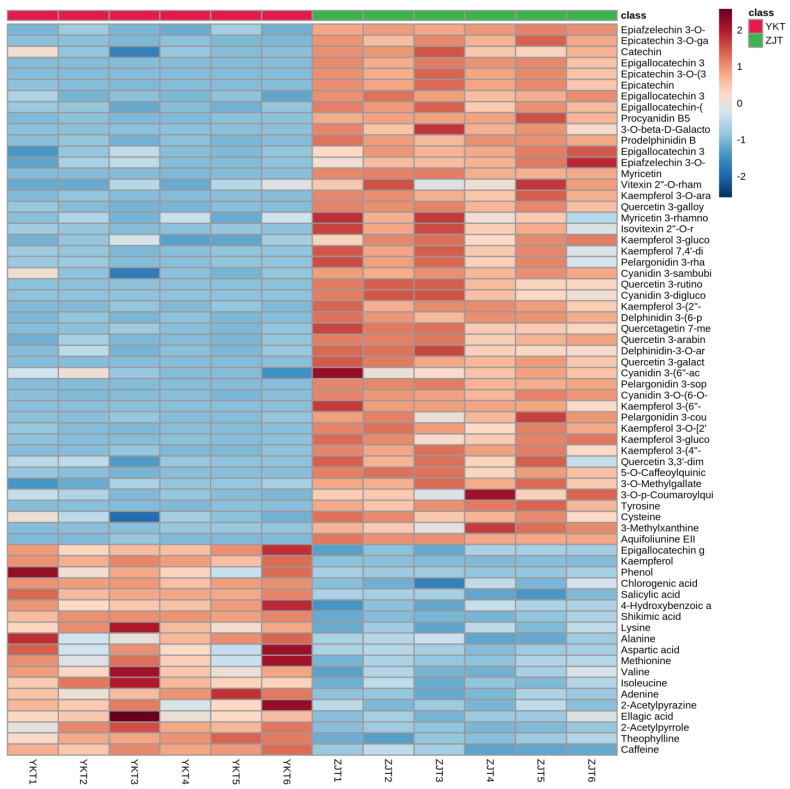
Heat map representing metabolites levels in ZJT and YKT. Red and blue boxes represent values that were higher and lower than the mean value, respectively.

Variables with VIP > 1, *p* < 0.05, fold change > 1.5 were selected as potential differential metabolites, which were identified by comparing RT, m/z value with corresponding published data in literature or databases. A total for 66 metabolites was tentatively identified, which including 8 flavan-3-ols, 6 proanthocyanins, 28 flavonol and flavone glycosides, 8 phenolic acids, 8 amino acids and 8 alkaloids summarized in Table 1. A heat map based on the 66 differentially abundant metabolites (Figure 2) provided a comprehensive overview of the differences in the metabolite contents between ZJT and YKT.

#### 3.2.1. Flavan-3-Ols

Flavan-3-ols are considered the main compounds that account for the antioxidant activity of tea [36]. Total catechins had almost same content in both ZJT and YKT teas (Table S1) which agreed with Yang et al. [15]. Wang et al. isolated and identified some catechins such as epigallocatechin 3-*O*-*p*-coumaroate, epigallocatechin-3-O-ferulate, (-)-epicatechin (EC), (-)-epigallocatechin (EGC), (-)-epicatechin-3-*O*-gallate (ECG), (-)-epigallocatechin-3-*O*-gallate (EGCG), (-)-epigallocatechin-3-*O*-(3′-O-methylgallate), (-)-epicatechin-3-*O*-(3′-*O*-methylgallate) in ZJT [5]. Catechins including catechin (C), EC, ECG, epicatechin 3-*O*-(3-*O*-methylgallate), epigallocatechin 3-(3”-methylgallate) (EGCG 3”Me), epigallocatechin 3-*p*-coumaroate and epiafzelechin 3-*O*-gallate in ZJT are significantly higher in ZJT than that in YKT (Table 1). While epigallocatechin gallate (EGCG) is low in ZJT compared with YKT.

#### 3.2.2. Proanthocyanins

**Figure 3:**
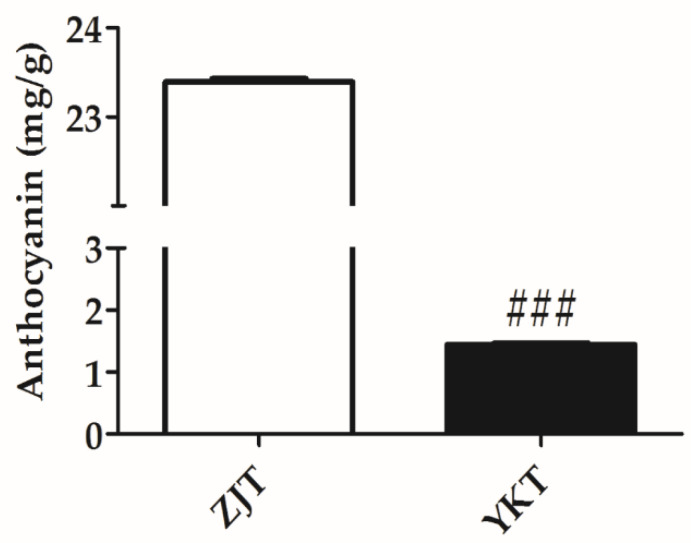
Concentration of anthocyanin in ZJT and YKT (mg/g) (*n* = 3, mean ± standard error of the mean (SEM)), ### *p* < 0.001, compared to ZJT.

Proanthocyanins are important flavonoids, which are dimers or polymers in the two or more different types of flavan-3-ol derivatives. Our data revealed that there were significantly differences in proanthocyanins contents between these two type teas, and ZJT contained much higher levels of proanthocyanins, such as procyanidin B5, prodelphinidin B and 3-O-beta-D-galactopyranosylproanthocyanidin A5′ compared with YKT (Table 1). C and EC were mainly precursors for proanthocyanin synthesis in some plants [7], which are both higher levels in ZJT than YKT (Table 1). Additionally, epigallocatechin 3-*O*-gallate-(4β->6)-epicatechin and epiafzelechin 3-*O*-gallate-(4beta->6)-epigallocatechin, the metabolites of epigallocatechin-(4beta->8)-epicatechin 3-*O*-gallate, also exhibit the two- to nine-fold increase in ZJT than that in YKT (Figure 3).

#### 3.2.3. Flavonol and Flavone Glycosides

Flavonol and flavone glycosides are a large family of compounds that possess bioactive properties. ZJT has 27 metabolites of flavonol and flavone glycosides exhibiting richer abundance than that in YKT, including myricetin, kaempferol glycosides (kaempferol 3-glucoside-7-xyloside, kaempferol 3-(2″-hydroxypropionylglucoside)-4′-glucoside, kaempferol 3-*O*-arabinoside, kaempferol 3-(4″-acetyl-6″-*p*-coumarylglucoside), kaempferol 7,4′-dirhamnoside, kaempferol 3-(6″-ferulylglucoside), kaempferol 3-*O*-[2″-(4‴-acetylrhamnosyl)-6″-glucosyl], kaempferol 3-glucoside), quercetin glycosides (quercetin 3-galloylglucosyl-arabinofuranoside, quercetin 3-rutinoside-4′-glucoside, quercetagetin 7-methylether 6-glucoside, quercetin 3-arabinoside, quercetin 3-galactoside, quercetin 3,3′-dimethylether 4′-glucoside), myricetin, vitexin glycosides (vitexin 2″-*O*-rhamnoside-4‴-acetate) and isovitexin glycosides (isovitexin 2″-*O*-rhamnoside) (Table 1). Only one metabolite kaempferol is lower in ZJT than YKT (Figure 2).

Anthocyanins are water-soluble natural pigments widely distributed in terrestrial plants [37]. As shown in Table 1, nine anthocyanins were identified in the ZJT, and their relative abundance were found significantly richer than that in YKT. These were putatively identified as cyanidin 3-diglucoside 5-glucoside, cyanidin 3-*O*-(6-*O*-*p*-coumaroyl) glucoside, cyanidin 3-sambubioside, cyanidin 3-(6″-acetylglucoside)-5-glucoside, delphinidin 3-(6-*p*-coumaroyl) galactoside, delphinidin-3-*O*-arabinoside, pelargonidin 3-sophoroside 5-glucoside, pelargonidin 3-coumarylglucoside-5-acetylglucoside, pelargonidin 3-rhamnoside 5-glucoside (Table 1) [38]. In terms of MS intensities, cyanidin 3-diglucoside 5-glucoside and pelargonidin 3-sophoroside 5-glucoside exhibited the 2579 and 63-fold higher in ZJT than that in YKT. Quantitative analysis using acid-alcohol method showed absolutely higher concentrations of total anthocyanins in ZJT (Figure 3), which was similar to the metabolomics results. Our results indicated that most of the anthocyanins were not synthesized at all in the YKT.

#### 3.2.4. Amino Acids and Phenolic Acids

Regarding free amino acids, our results indicated that ZJT possessed relatively higher amounts of tyrosine and cysteine (Table 1). Lysine, alanine, aspartic acid, methionine, valine and isoleucine have lower levels in ZJT than YKT (Figure 2) in this study.

Additionally, phenolic acids in ZJT and YKT extracts differed greatly. Higher levels of caffeoylquinic acid, methyl gallate and coumaroylquinic acid were observed in ZJT (Table 1) and phenol, chlorogenic acid, ellagic acid, salicylic acid, 4-hydroxybenzoic acid and shikimic acid were found in lower contents in ZJT than YKT (Figure 2).

#### 3.2.5. Alkaloids

Alkaloids, mainly caffeine and theobromine, has been extensively investigated in tea plants [13,39]. In this study, 3-methylxanthine and aquifoliunine EII were higher levels in ZJT than YKT (Table 1), while adenine, 2-acetylpyrazine, 2-acetylpyrrole, theophylline and caffeine were lower in ZJT than YKT (Figure 2). It was reported that genotypic factors had many effects on the caffeine content of tea which might be the reason why ZJT and YKT had different caffeine content [40].

### 3.3. Metabolic Pathway Analysis of ZJT

**Figure 4:**
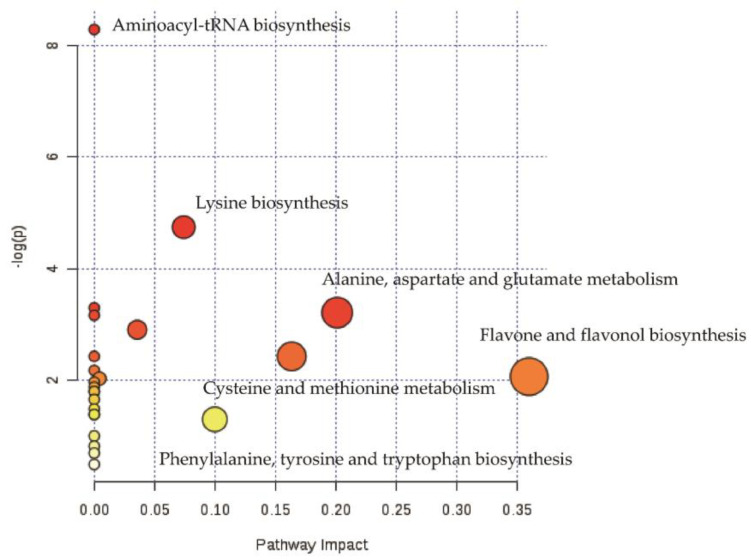
Pathway analysis of ZJT and YKT. The *y*-axis (−log (*p*-value)) and *x*-axis represents the significance of the pathway and pathway impact between ZJT and YKT, respectively.

Pathway analysis of differential metabolites helps us to understand the mechanism of metabolic pathway changes in differential groups. Figure 4 showed the enrichment analysis of the metabolic pathway of ZJT. Data in the enrichment metabolic pathway were plotted with the significance of the pathway (−log (*p*-value)) as the ordinate, using the metabolic pathway impact as abscissa. Our data showed that the differential metabolites in ZJT were mainly enriched in the two pathways, flavonoid biosynthesis and flavone/flavonol biosynthesis when compared against YKT. The most influential metabolic pathway had a pathway impact > 0.35 and −log (*p*-value) > 2.

**Figure 5:**
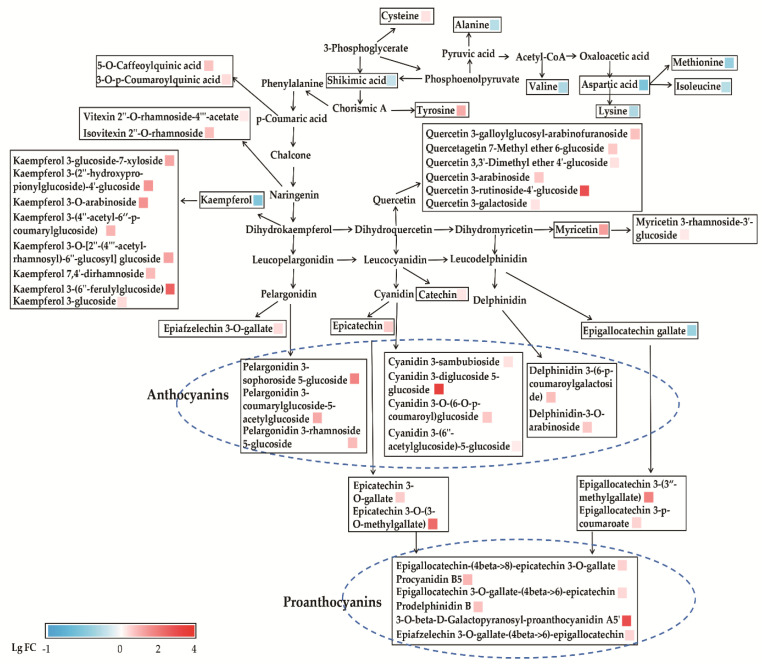
Metabolic pathways of flavone and flavonol biosynthesis and amino acid biosynthesis in ZJT and YKT. Red (LgFC > 0) and blue (LgFC < 0) boxes represent values that were higher and lower than the mean value, respectively.

The pathways of flavone and flavonol biosynthesis and amino acid biosynthesis are further analyzed and mapped in Figure 5. It showed that a set of the metabolites in the biosynthesis of flavone and flavonol were higher in ZJT than YKT except for kaempferol and EGCG. As shown in the Figure 5, ZJT has significantly higher proanthocyanins, which including epigallocatechin-(4beta->8)-epicatechin 3-*O*-gallate, procyanidin B5, epigallocatechin 3-*O*-gallate-(4beta->6)-epicatechin, 3-*O*-beta-D-galactopyranosylproanthocyanidin A5′, prodelphinidin B and epiafzelechin 3-*O*-gallate-(4beta->6)-epigallocatechin. Furthermore, in the flavone and flavonol biosynthesis pathway, the production of kaempferol glycosides, quercetin glycosides, myricetin glycosides and anthocyanins was also increased on a large scale (Figure 5). Free amino acids play important roles in the determination of tea testers and are also involved in the formation of tea aroma. Amino acid contents in tea are considered to be closely correlated to tea varieties [41,42]. Our results showed that metabolites in the amino acid biosynthesis pathway, such as shikimic acid, alanine, valine, and aspartic acid, are all abundant in YKT. Our data indicated that these rich amino acid may contribute to specific aroma and tester formation in ZJT.

## 4. Discussion

ZJT is a representative purple tea. In this study, a UHPLC-Orbitrap-MS/MS based non-targeted metabolomics approach combined with a chemometrics analysis was used to investigate the metabolite profiling and to identify characteristic compounds of ZJT against green tea YKT. A total of 66 metabolites were characterized, including 8 flavan-3-ols, 6 proanthocyanins, 28 flavonol and flavone glycosides, 8 phenolic acids, 8 amino acids and 8 alkaloids.

In our study, total catechins had almost same content in both teas by HPLC analysis (Table S1) which agreed with Yang et al. [15]. While Yan et al. reported that ZJT had lower total catechins than YKT [43]. This difference might be due to the different harvest season of ZJT and YKT in Yan’s work. A previous paper reported that the total catechin concentrations were significantly higher in tea plants harvested in autumn than in those harvested in spring [44]. However, the ZJT and YKT of this study were collected in the same tea garden and manufactured at the same time. Our data revealed that the concentrations of 6 types of catechin showed similar levels in trends between ZJT and YKT. It is worth noting that relative abundance of 3” methylated EGCG (EGCG 3”Me) in ZJT has approximate 66-folde higher than that in YKT by comparing mass intensity (Table 1). Epigallocatechin gallate (EGCG) is low in ZJT compared with YKT. This might be due to some parts of EGCG being modified to form EGCG 3”Me in the ZJT tea tree. It was reported that the content of EGCG 3”Me in fresh ZJT leaves was as high as 1.05% [45]. Other Yunnan large-leaf teas (*Camellia sinensis var. assamica*) including YKT tree leaves do not generally contain EGCG 3”Me. Kurita et al. reported that EGCG 3”Me exhibited significant hypotensive effects in clinical trials [46]. In addition, a paper reported that the bioavailability of EGCG 3”Me is superior to EGCG [47]. Our data suggested that high concentration of EGCG 3”Me may have potential benefits in ZJT.

Anthocyanins are generally responsible for the red, blue, and purple pigments in plant leaves. The sufficient of anthocyanins could dominate the green color of chlorophylls, leaf coloration then depends mainly on anthocyanins [48]. In this study, ZJT has more flavonol and flavone glycosides than YKT. Similar results have also been reported by Yang et al. [15]. The relative abundance of 9 anthocyanins identified in the ZJT was found to be significantly richer than that in YKT. The total amount of anthocyanins in ZJT was also significantly higher than that of YKT (Figure 3). Therefore, anthocyanins might overlap with the chlorophyll to form the more purple color in ZJT.

The flavone and flavonol biosynthesis and amino acid biosynthesis pathway are further mapped in Figure 5. As shown, chorismic acid is a key intermediate for p-coumaric acid and tyrosine synthesis. Tyrosine is the precursor of coumaroyl CoA, which promote the production of flavonoids [49]. In this pathway, the downstream metabolites caffeoylquinic acid, coumaroylquinic acid and tyrosine were significantly higher in ZJT. Phenylalanine is catalyzed by a series of enzymes such as chalcone synthase (*CHS*), chalcone isomerase (*CHI*), dihydroflavonol 4-reductase (*DFR*), anthocyanin synthetase (*ANS*), UDP-glucosyl transferase (*UGT*) to produce dihydrokaempferol, and it is also the common precursor for favan-3-ols, proanthocyanins, flavonol and flavone glycosides [43,50,51]. It was reported that the abundances of anthocyanin synthesis-related enzymes, such as *CHS*, *CHI*, *DFR*, *ANS* and *UGT* in the purple leaves were all significantly higher than those in the green leaves. The increase expression of anthocyanin synthesis-related enzymes contributes to anthocyanin accumulation [43,52,53]. Furthermore, dihydrokaempferol is catalyzed by *ANS* to form colorless leucopelargonidin, which is the precursor of pelargonidin, and the pelargonidin combines with glycosides under the catalysis of *UGT* to form stable pelargonidin glycosides (pelargonidin 3-sophoroside 5-glucoside, pelargonidin 3-coumarylglucoside-5-acetylglucoside, pelargonidin 3-rhamnoside 5-glucoside). Our results showed that pelargonidin glycosides were higher in ZJT than YKT. In addition, dihydrokaempferol produces kaempferol, which in combination with glycosides forms kaempferol glycosides (kaempferol 3-glucoside-7-xyloside, kaempferol 3-(2″-hydroxypropionylglucoside)-4′-glucoside, kaempferol 3-*O*-arabinoside, kaempferol 3-(4″-acetyl-6″-*p*-coumarylglucoside), kaempferol 7,4′-dirhamnoside, kaempferol 3-(6″-ferulylglucoside), kaempferol 3-*O*-[2″-(4‴-acetylrhamnosyl)-6″-glucosyl], kaempferol 3-glucoside). Thus, leucopelargonidin forms anthocyanins in the flavonoid biosynthesis pathway in ZJT. In the study of Li et al., increased acetyl-CoA may contribute to anthocyanin accumulation through the increase of cytosolic acetyl-coenzyme A carboxylase activity in the purple leaves of ZJT, indicating that glycolysis may provide more intermediates as substrates for promoting anthocyanin accumulation [50]. In particular, dihydroquercetin is a common direct precursor for both quercetin and leucocyanidin. Consequently, it is reasonable that large amounts of quercetin-based flavonol glycosides accumulated in ZJT should enhance the production of cyanidin-based anthocyanins. The extensively enhanced flavonoid pathway affirmatively provides more available precursors for flavanol biosynthesis in ZJT. Consequently, the polycondensation of proanthocyanin monomers might be further promoted in ZJT.

The other two pathways catalyze the formation of dihydroquercetin and dihydromyricetin under a series of enzymes’ action to produce flavonoid glycosides and anthocyanins, whereas most catechins, quercetin glycosides, myricetin glycosides, cyanidin glycosides and delphinidin glycosides are produced by the similar enzymatic reaction. A previous paper reported that transcription levels of anthocyanin synthesis-related genes, including *PAL*, *CHS*, *CHI, DFR* and *ANS* in ZJT, were high due to activation of the transcription factors *bHLH* and *HY5* which resulted in enhanced anthocyanin synthesis in purple leaves of ZJT [43]. Furthermore, the amino acids biosynthesis pathway analysis showed that shikimic acid, alanine, valine, and aspartic acid were all abundance in YKT (Figure 5). Those two pathways all contribute to the special flavor and color of ZJT [43,50,51].

The chemical profiles and characteristic metabolites between Zijuan purple tea and Yunkang green tea were obtained in specific conditions. One is that Zijuan and Yunkang cultivars were cultivated in the same tea garden of Institute of Tea Science, Yunnan Province Academy of Agricultural Sciences. The other is that ZJT and YKT were produced by standard steamed green tea protocol. Therefore, the present results cannot be generalized to other purple or green varieties of tea apart from the anthocyanin content.

## 5. Conclusions

The UHPLC-Orbitrap-MS/MS technique and chemometrics methods are powerful tools to comparatively analyze chemical profiles and characteristic metabolites between Zijuan purple tea and Yunkang green tea. Based on chemical profiles, ZJT and YKT are greatly differentiated even if they are made from leaves and buds collected from the same variety (*Camellia sinensis var. assamica*). Catechins, proanthocyanins, flavonol and flavone glycosides, phenolic acids, amino acids and alkaloids are major differential metabolites between ZJT and YKT. In addition, pathway analysis revealed that ZJT might generate anthocyanins and proanthocyanins through the flavonol and flavone glycosides. The increase of a set of anthocyanins and proanthocyanins all contribute to the specific purple color of ZJT. This study presented the comprehensive chemical profiling and the characterized metabolites of ZJT. These results also provided chemical evidence for the potential health functions of ZJT.

## Figures and Tables

**Table 1:** Differential metabolites in ZJT and YKT samples by metabolomics analysis based on ultra-high performance liquid chromatography combined with Orbitrap mass spectrometry (UHPLC-Orbitrap-MS/MS).

Compound Name	RT (min)	Detected Mass	Theoretical Mass	Adduct	Formula	MS/MS Fragment	VIP	*p*-Value	|Log_2_ FC|	FC
**Flavan-3-ols**										
Epiafzelechin 3-*O*-gallate	4.30	426.0945	426.0951	[M-H_2_O-H]-	C_22_H_18_O_9_	169, 125	1.38	2.00 × 10^−9^	1.30	2.46
Epicatechin 3-*O*-gallate	4.49	442.0925	442.0905	[M-H_2_O-H]-	C_22_H_18_O_10_	123, 139, 153	1.37	3.00 × 10^−8^	1.97	3.91
Catechin *	4.60	290.0785	290.0790	[M+H]+	C_15_H_14_O_6_	95,97,109,123,125	1.23	2.00 × 10^−4^	0.83	1.78
Epigallocatechin gallate *	5.02	458.0831	458.0849	[M+H]+	C_22_H_18_O_11_	139, 151, 289	1.45	4.00 × 10^−3^	1.31	0.40
Epigallocatechin 3-(3”-methylgallate)	5.96	472.1006	472.1006	[M-H]-	C_23_H_20_O_11_	139, 140, 167	1.38	4.00 × 10^−9^	6.06	66.66
Epicatechin 3-*O*-(3-*O*-methylgallate)	7.32	456.1055	456.1056	[M-H]-	C_23_H_20_O_10_	109, 124, 168, 183, 289	1.38	2.00 × 10^−8^	7.61	195.68
Epicatechin *	7.34	290.0793	290.0790	[M-H]-	C_15_H_14_O_6_	123, 139, 147, 207	1.37	2.00 × 10^−8^	3.57	11.87
Epigallocatechin 3-*p*-coumaroate	8.12	452.1109	452.1107	[M-H]-	C_24_H_20_O_9_	196, 255	1.37	5.00 × 10^−8^	1.60	3.03
**Proanthocyanins**										
Epigallocatechin-(4beta->8)-epicatechin 3-*O*-gallate	4.27	746.1481	746.1483	[M+H]+	C_37_H_30_O_17_	305, 443, 579	1.36	3.00 × 10^−7^	1.73	3.32
Procyanidin B5	5.25	578.1430	578.1424	[M-H_2_O-H]-	C_30_H_26_O_12_	289, 469	1.37	8.00 × 10^−8^	3.05	8.29
3-*O*-beta-D-Galactopyranosylproanthocyanidin A5′	5.50	738.1771	738.1796	[M+Na]+	C_36_H_34_O_17_	195, 455, 723	1.31	8.00 × 10^−6^	9.93	976.56
Prodelphinidin B	6.01	610.1335	610.1323	[M+H]+	C_30_H_26_O_14_	309, 757	1.38	2.00 × 10^−9^	2.46	5.49
Epigallocatechin 3-*O*-gallate-(4beta->6)-epicatechin 3-*O*-gallate	6.67	898.1590	898.1593	[M+H]+	C_44_H_34_O_21_	433, 741	1.32	5.00 × 10^−6^	1.39	2.62
Epiafzelechin 3-*O*-gallate-(4beta->6)-epigallocatechin 3-*O*-gallate	7.41	882.1608	882.1643	[M+H]+	C_44_H_34_O_20_	271, 441, 591	1.25	8.00 × 10^−5^	1.53	2.89
**Flavonol and flavone glycosides**										
Myricetin	3.65	318.0383	318.0376	[M-H_2_O-H]-	C_15_H_8_O_7_	125, 169, 241	1.39	9.00 × 10^−10^	4.31	19.85
Vitexin 2″-*O*-rhamnoside-4‴-acetate	3.77	620.1727	620.1741	[M-H_2_O-H]-	C_29_H_32_O_15_	339, 449, 493	1.16	9.00 × 10^−4^	0.69	1.61
Kaempferol 3-*O*-arabinoside	4.05	418.0891	418.0900	[M+H]+	C_20_H_18_O_10_	211, 401	1.37	7.00 × 10^−8^	5.07	33.57
Quercetin 3-galloylglucosyl-arabinofuranoside	4.32	748.1520	748.1487	[M-H]-	C_33_H_32_O_20_	229, 285	1.38	8.00 × 10^−9^	2.46	5.50
Myricetin 3-rhamnoside-3′-glucoside	4.95	626.1495	626.1483	[M-H]-	C_27_H_30_O_17_	178, 316	1.05	5.00 × 10^−3^	0.72	1.64
Isovitexin 2″-*O*-rhamnoside	4.95	578.1621	578.1636	[M+H]+	C_27_H_30_O_14_	247, 291, 409, 427	1.24	1.00 × 10^−4^	2.67	6.36
Kaempferol 3-glucoside	5.27	448.1007	448.1006	[M-H_2_O-H]-	C_21_H_20_O_11_	145, 301, 377	1.29	2.00 × 10^−5^	1.34	2.52
Kaempferol 7,4′-dirhamnoside	5.50	578.1629	578.1636	[M-H]-	C_27_H_30_O_14_	245, 289	1.26	6.00 × 10^−5^	2.75	6.72
Pelargonidin 3-rhamnoside 5-glucoside	5.50	579.1722	579.1714	[M-H]-	C_27_H_31_O_14_	154, 245, 289, 469	1.25	1.00 × 10^−4^	2.79	6.90
Cyanidin 3-sambubioside	5.63	581.1510	581.1506	[M-H]-	C_26_H_29_O_15_	419, 435, 449, 458	1.28	3.00 × 10^−5^	0.99	1.99
Quercetin 3-rutinoside-4′-glucoside	5.90	772.2045	772.2062	[M-H]-	C_33_H_40_O_21_	151, 255, 271, 301, 300	1.31	7.00 × 10^−6^	9.73	849.64
Cyanidin 3-diglucoside 5-glucoside	5.90	773.2145	773.2140	[M-H]-	C_33_H_41_O_21_	277, 513	1.30	1.00 × 10^−5^	11.33	2579.02
Kaempferol 3-(2″-hydroxypropionylglucoside)-4′-glucoside	5.94	682.1734	682.1745	[M-H_2_O-H]-	C_30_H_34_O_18_	213, 249, 327	1.36	3.00 × 10^−7^	4.87	29.33
Delphinidin 3-(6-*p*-coumaroylgalactoside)	6.01	611.1379	611.1401	[M+H]+	C_30_H_27_O_14_	138, 331, 409	1.38	2.00 × 10^−9^	2.65	6.29
Quercetagetin 7-methylether 6-glucoside	6.04	494.1060	494.1060	[M-H]-	C_22_H_22_O_13_	163, 319, 337	1.30	1.00 × 10^−5^	2.04	4.10
Quercetin 3-arabinoside	6.11	434.0853	434.0849	[M-H]-	C_20_H_18_O_11_	343, 313	1.36	1.00 × 10^−7^	2.15	4.43
Delphinidin-3-*O*-arabinoside	6.12	435.0933	435.0927	[M-H]-	C_20_H_19_O_11_	165, 289, 341	1.26	8.00 × 10^−5^	2.11	4.33
Quercetin 3-galactoside	6.14	464.0944	464.0955	[M+H]+	C_21_H_20_O_12_	138, 303	1.08	3.00 × 10^−3^	0.91	1.88
Cyanidin 3-(6″-acetylglucoside)-5-glucoside	6.31	652.1641	652.1639	[M-H_2_O-H]-	C_29_H_32_O_17_	207, 315, 515	1.08	3.00 × 10^−3^	0.63	1.55
Pelargonidin 3-sophoroside 5-glucoside	6.32	757.2184	757.2191	[M+H]+	C_33_H_41_O_20_	287, 433, 595	1.39	5.00 × 10^−10^	5.98	63.09
Cyanidin 3-*O*-(6-*O*-*p*-coumaroyl)glucoside	6.46	594.1373	594.1373	[M+H]+	C_30_H_26_O_13_	166, 273, 424, 442, 527	1.39	2.00 × 10^−10^	2.17	4.49
Kaempferol 3-(6″-ferulylglucoside)	6.51	624.1464	624.1479	[M+H]+	C_31_H_28_O_14_	317, 165, 203	1.33	2.00 × 10^−6^	8.49	360.45
Pelargonidin 3-coumarylglucoside-5-acetylglucoside	6.52	782.2061	782.2058	[M-H_2_O-H]-	C_38_H_38_O_18_	457, 461, 337	1.31	1.00 × 10^−5^	3.73	13.26
Kaempferol 3-*O*-[2″-(4‴--acetyl-rhamnosyl)-6″-glucosyl] glucoside	6.53	798.2216	798.2219	[M+H]+	C_35_H_42_O_21_	519, 524	1.36	2.00 × 10^−7^	4.01	16.15
Kaempferol 3-glucoside-7-xyloside	6.83	580.1452	580.1428	[M+H]+	C_26_H_28_O_15_	271, 396	1.33	2.00 × 10^−6^	3.93	15.27
Kaempferol 3-(4″-acetyl-6″-*p*-coumarylglucoside)	8.14	636.1456	636.1479	[M-H_2_O-H]-	C_29_H_32_O_16_	313, 465, 483	1.36	2.00 × 10^−7^	3.16	8.93
Quercetin 3,3′-dimethyl ether 4′-glucoside	8.58	510.1369	510.1373	[M-H_2_O-H]-	C_23_H_26_O_13_	285, 442, 599	1.18	5.00 × 10^−4^	1.04	2.05
Kaempferol	8.90	286.0465	286.0477	[M+H]+	C_15_H_10_O_6_	121, 165, 241	1.55	4.00 × 10^−3^	1.80	0.29
**Phenolic acids**										
Phenol	0.74	94.0414	94.0419	[M+H]+	C_6_H_6_O	62, 73, 82	1.23	4.00 × 10^−3^	1.24	0.42
5-*O*-Caffeoylquinic acid	1.51	354.0955	354.0951	[M-H]+	C_16_H_18_O_9_	103, 175	1.36	2.00 × 10^−7^	2.33	5.02
Chlorogenic acid	2.42	354.0933	354.0951	[M+Na]+	C_16_H_18_O_9_	93, 135, 173, 191	1.47	4.00 × 10^−3^	0.71	0.61
Salicylic acid	4.24	138.0309	138.0317	[M+H]+	C_7_H_6_O_3_	56, 65, 116, 139, 140	1.52	4.00 × 10^−3^	1.44	0.37
4-Hydroxybenzoic acid	5.02	138.0312	138.0317	[M+H]+	C_7_H_6_O_3_	56, 111, 116, 139, 140	1.40	4.00 × 10^−3^	0.69	0.62
3-*O*-Methylgallate	7.35	184.0376	184.0372	[M-H]-	C_8_H_8_O_5_	124, 139, 168	1.33	3.00 × 10^−6^	0.97	1.97
3-*O*-*p*-Coumaroylquinic acid	7.54	338.1000	338.1002	[M-H_2_O-H]-	C_16_H_18_O_8_	93, 119, 173, 191	1.15	1.00 × 10^−3^	1.31	2.48
Shikimic acid	9.49	174.0521	174.0528	[M-H_2_O-H]-	C_7_H_10_O_5_	61, 67, 93, 173	1.33	4.00 × 10^−3^	0.68	0.62
**Amino acids**										
Lysine	0.63	146.1050	146.1055	[M+H]+	C_6_H_14_N_2_O_2_	72, 84, 128, 130	1.36	4.00 × 10^−3^	1.01	0.50
Alanine	0.72	89.0472	89.0477	[M+H]+	C_3_H_7_NO_2_	68, 77	1.27	4.00 × 10^−3^	0.88	0.54
Aspartic acid	0.74	133.0371	133.0375	[M+H]+	C_4_H_7_NO_4_	74, 88, 102, 116	1.15	4.00 × 10^−3^	1.84	0.28
Methionine	1.04	149.0503	149.0510	[M+H]+	C_5_H_11_NO_2_S	102, 131	1.23	4.00 × 10^−3^	1.37	0.39
Valine	1.05	117.0785	117.0790	[M+H]+	C_5_H_11_NO_2_	58, 59, 118, 119	1.32	4.00 × 10^−3^	1.04	0.49
Isoleucine	1.92	131.0939	131.0946	[M+H]+	C_6_H_13_NO_2_	69, 72, 86, 90	1.39	4.00 × 10^−3^	0.89	0.54
Tyrosine	2.76	181.1882	181.1885	[M-H]-	C_9_H_11_NO_3_	91, 119, 123, 136, 165	1.37	7.00 × 10^−8^	3.57	11.84
Cysteine	5.63	121.1586	121.1582	[M-H]-	C_3_H_7_NO_2_S	74, 100, 98	1.22	2.00 × 10^−4^	0.90	1.87
**Alkaloids**										
Adenine	0.63	135.0556	135.0545	[M-H]-	C_5_H_5_N_5_	119	1.28	4.00 × 10^−3^	0.84	0.56
2-Acetylpyrazine	1.04	122.0475	122.0480	[M+H]+	C_6_H_6_N_2_O	80, 95, 96, 123	1.28	4.00 × 10^−3^	1.91	0.27
3-Methylxanthine	1.15	166.0487	166.0491	[M+H]+	C_6_H_6_N_4_O_2_	120	1.28	4.00 × 10^−5^	3.17	9.01
Ellagic acid	3.86	302.0048	302.0063	[M+H]+	C_14_H_6_O_8_	57, 275, 285, 303	1.20	4.00 × 10^−3^	1.71	0.31
2-Acetylpyrrole	4.27	109.0526	109.0528	[M+Hac-H]-	C_6_H_7_NO	67, 83	1.29	4.00 × 10^−3^	1.42	0.37
Theophylline	6.27	180.0641	180.0647	[M-H_2_O+H]-	C_7_H_8_N_4_O_2_	135, 146, 161, 164	1.50	4.00 × 10^−3^	0.62	0.65
Aquifoliunine EII	6.32	721.2591	721.2582	[M+K]+	C_34_H_43_NO_16_	231, 273, 411	1.39	2.00 × 10^−10^	4.82	28.31
Caffeine *	9.54	194.0800	194.0804	[M-H_2_O-H]-	C_8_H_10_N_4_O_2_	85, 93	1.30	4.00 × 10^−3^	0.59	0.66

Note: Fold change (FC) is based on comparing the mass intensity of metabolites between ZJT and YKT; metabolite marked with * is confirmed by standards.

## Data Availability

No new data were created or analyzed in this study. Data sharing is not applicable to this article.

## Supplementary Materials

The following are available online at https://www.mdpi.com/article/10.3390/foods10051070/s1, Figure S1: The representative total ion chromatogram (TIC) of UHPLC-Orbitrap-MS/MS in ZJT. (A): positive ion mode, (B): negative ion mode. Table S1: Contents of catechins and amino acids in ZJT and YKT (%) (*n* = 3, mean ± SEM).
