# Supplementary material for: Analysis of Differentiated Chemical Components between Zijuan Purple Tea and Yunkang Green Tea by UHPLC-Orbitrap-MS/MS Combined with Chemometrics
Source: Foods. 2021 May 12;10(5):1070. doi: 10.3390/foods10051070 (PMC8151513; doi:10.3390/foods10051070)
Supplement: Supplementary file 1 [file foods-10-01070-s001.zip › foods-1175896-supplementary.pdf]

Supplrmentary Materials

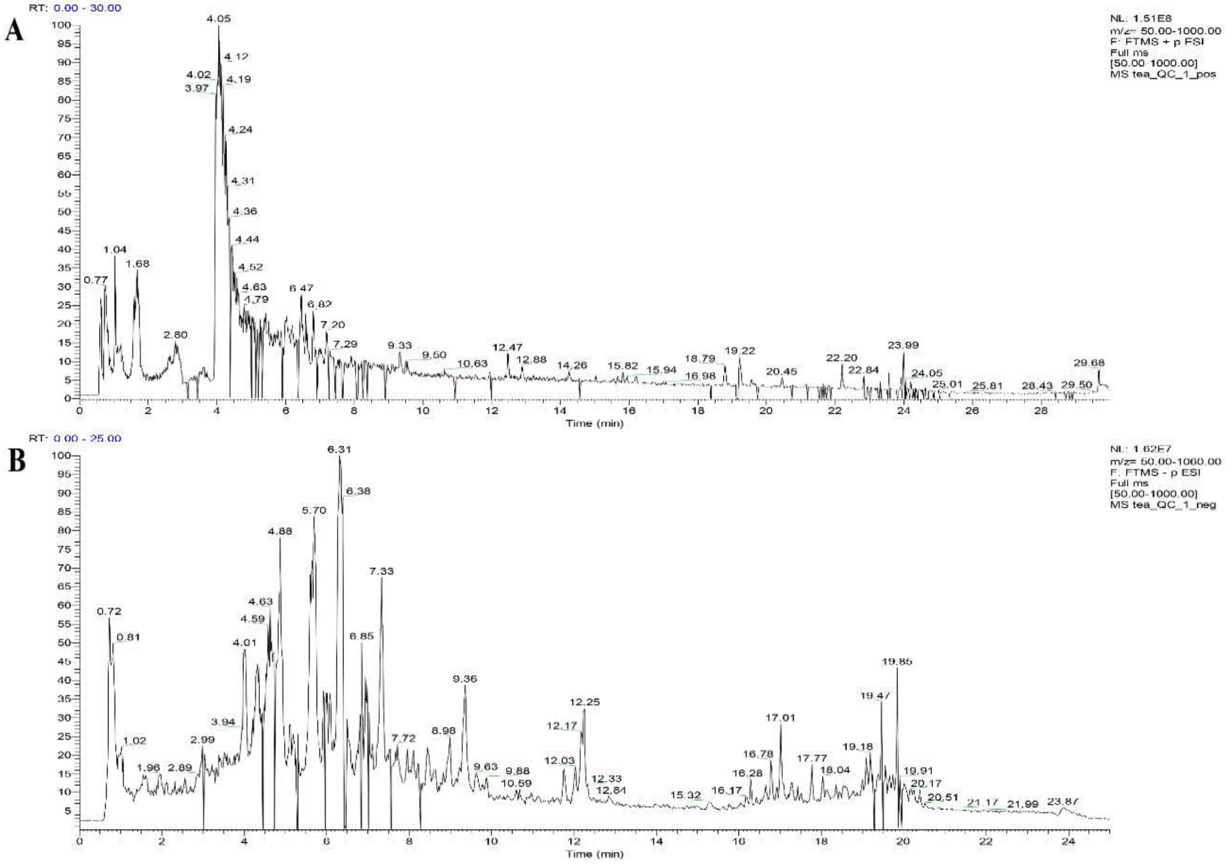

Figure 1. The total ion chromatogram (TIC) of UHPLC-Orbitrap-MS/MS in ZJT. (**A**): positive ion mode, (**B**): negative ion mode.

Table S1. Contents of catechins and caffeine in ZJT and YKT (%) (n = 3, mean ± SEM).

Sample	YKT	ZJT
EGC	1.439 ± 0.093	1.891 ± 0.164
C	0.253 ± 0.065	0.577 ± 0.109 #
EC	0.668 ± 0.039	1.229 ± 0.100 ##
EGCG	12.500 ± 1.048	7.194 ± 0.709 **
ECG	3.282 ± 0.273	3.426 ± 0.343
Caffeine	4.051 ± 0.307	3.792 ± 0.320
Total catechin	18.370 ± 1.524	14.400 ± 1.425

Note: EGC, ()-epigallocatechin; C, (+)-Catechin; EC, (-)-epi-catechin; EGCG, ()-epigallocatechin gallate; ECG, ()-epicatechin gallate; # $p < 0.05$, ## $p < 0.01$, less content than ZJT; ** $p < 0.01$, more content than ZJT.
